# An italian multicenter triple-blind randomized controlled trial on photobiomodulation after third molar extraction (BIOSTOTT). A study of Italian society for laser in dentistry (SILO)

**DOI:** 10.4317/medoral.27834

**Published:** 2025-11-22

**Authors:** Ilaria Giovannacci, Umberto Romeo, Francesco Spadari, Matteo Biasotto, Monica Pentenero, Giuseppe Pedrazzi, Aurora Andrea Venuti, Marco Meleti, Gianluca Tenore, Daniele Pergolini, Massimo Porrini, Moreno Bosotti, Giulia Ottaviani, Katia Rupel, Matteo Val, Paolo Vescovi

**Affiliations:** 1 Oral Medicine and Oral Laser Surgery Unit, Department of Medicine and Surgery, University of Parma, Via Gramsci 14, 43125 Parma, Italy; 2 Department of Oral Sciences and Maxillofacial Surgery, Sapienza University of Rome, Rome, Italy; 3 Department of Biomedical, Surgical and Dental Sciences, University of Milan; Maxillo-Facial and Odontostomatology Unit, Ospedale Policlinico, Fondazione IRCCS Ca' Granda, Milan, Italy; 4 Division of Oral Medicine, Department of Medical, Surgical and Health Sciences, University of Trieste, Trieste, Italy; 5 Oral Medicine and Oral Oncology Unit, Department of Oncology, University of Turin, Turin, Italy; 6 Department of Medicine and Surgery, Unit of Neuroscience, Plesso Biotecnologico Integrato, University of Parma, 43125 Parma, Italy; 7 Interdepartmental Center of Robust Statistics (Ro.S.A.), University of Parma, 43125 Parma, Italy

## Abstract

**Background:**

Surgical extraction of third molars is a common oral and maxillo-facial surgery procedure frequently associated with postoperative complications, such as pain, swelling, and trismus. Photobiomodulation (PBM), also known as Low-Level Laser Therapy (LLLT), involves the use of low-intensity laser to promote tissue healing, reduce inflammation, and relieve pain. This study evaluated the effectiveness of PBM following surgical extraction of mandibular third molars.

**Material and Methods:**

This was a prospective, multicenter, randomized, triple-blind clinical trial conducted across five Italian centers. Seventy-nine patients were randomly assigned to a test group (PBM) or control group (no PBM). PBM was delivered immediately after surgery and on the following two days, using a multiband diode laser (445, 660 and 970nm). Primary outcomes were postoperative pain, facial swelling, and trismus. Secondary outcomes included health-related quality of life and analgesic consumption. Covariates included patient age, gender, the extracted tooth and the impaction classification according to Pell &amp; Gregory and Winter. The time of the surgical procedure, measured in seconds from incision to final suture, was also documented. Descriptive statistics were calculated for all variables. Normality was assessed using the Shapiro-Wilk test, and homogeneity of variances was evaluated using Levene's test. Between-group comparisons for continuous outcomes were performed using Student's t-test or the Mann-Whitney U test, depending on data distribution. Categorical vari-ables were analyzed with chi-square or Fisher's exact test. A p-value&lt; 0.05 was considered statistically significant.

**Results:**

No statistically significant differences were found between groups for any of the measured outcomes. However, trends favored the PBM group, particularly regarding reduced pain and improved quality of life.

**Conclusions:**

The study was powered to detect a large effect size (Cohen's d=0.8); therefore, the lack of statistical significance suggests that any true effect, if present, is likely smaller than this threshold. Further studies with larger sample sizes and standardized protocols are needed to explore smaller yet clinically relevant effects.

## Introduction

Extraction of impacted third molars is probably the most frequently performed procedure in oral and maxillofacial surgery (1 , 2). Its widespread indication is mainly attributable to two factors: The high prevalence of impacted third molars within the general population and the frequent occurrence of clinical symptoms associated with their persistence in the oral cavity. Impacted third molars are frequently associated with recurrent pericoronitis, abscesses, pulpal lesions, follicular cystic degeneration, or osteomyelitis, all complications that represent indications for surgical removal (3). In cases of asymptomatic impacted or retained third molars, the decision to proceed with prophylactic extraction remains one of the most challenging for dental professionals, particularly considering the potential morbidity associated with the procedure and the delicate balance between risks and benefits (4). As with any surgical procedure, extraction of impacted third molars carries a risk of intra- and post-operative complications. The most common postoperative complications include pain, edema, and trismus typically peaking between 12- and 48-hours post-surgery and gradually resolving within 5 to 7 days. Other potential complications include subcutaneous or intraoral mucosal hematoma, emphysema, and transient or permanent paresthesia of the lower lip (5 - 7). To improve patient outcomes and satisfaction, recent research has increasingly focused on pharmacological strategies and new technologies to optimize postoperative recovery. Among these, photobiomodulation therapy (PBM) has gained attention as a promising, noninvasive adjunctive approach for managing common postoperative complications (8 - 10). Also known as Low-Level Laser Therapy (LLLT), PBM exerts three main clinical effects: Promotion of wound healing, anti-inflammatory action, and pain relief. In vitro, animal and human studies have shown that LLLT stimulates DNA synthesis, cell proliferation (keratinocytes, fibroblasts, endothelial cells), collagen production, neoangiogenesis, macrophage activation, and wound contraction (11). Its analgesic action is mediated by enhanced -endorphins release, inhibition of C-fiber activity and bradykinin, as well as morphological neuronal changes that reduce mitochondrial membrane potential and temporarily block neural conduction (12 , 13). The anti-inflammatory response involves dose-dependent inhibition of cytokines such as IL-6, IL-10, TNF-, and MCP-1, leading to reduced vascular permeability, improved microcirculation, lymphatic drainage, and edema resolution (14 , 15). In dentistry, PBM has been applied in a wide range of clinical contexts, including the management of postoperative pain and inflammation following surgical procedures, the treatment of temporomandibular disorders (16), and the enhancement of bone and soft tissue healing in implantology and periodontology (17 - 19). It has also shown therapeutic potential in oral medicine, specifically for conditions such as oral mucositis (20), Oral Lichen Planus (21 , 22), and ulcerative lesions, like recurrent aphthous stomatitis (23). Moreover, it seems to be effective as an adjunctive modality in endodontic treatments (24) and the management of dentinal hypersensitivity (25). Although Low-Level Laser Therapy has been used to reduce postoperative swelling and trismus following third molar surgery, the reported outcomes remain controversial. The aim of the present study was to evaluate the effects of PBM on postoperative discomfort following surgical extraction of impacted mandibular third molars, with primary endpoints being reductions in postoperative pain, facial swelling, and trismus, and secondary endpoints assessing analgesic consumption and quality-of-life improvements.

## Material and Methods

1

**Figure 1:**
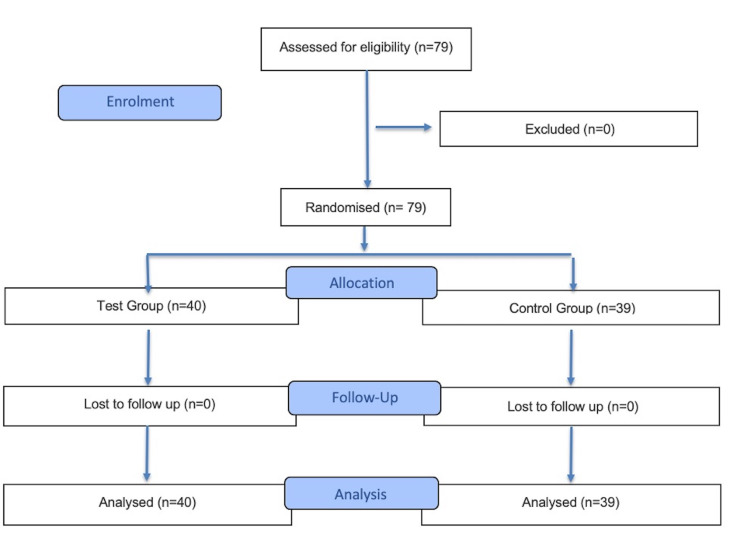
Flowchart of patient recruitment according to the CONSORT statement.

**Figure 2:**
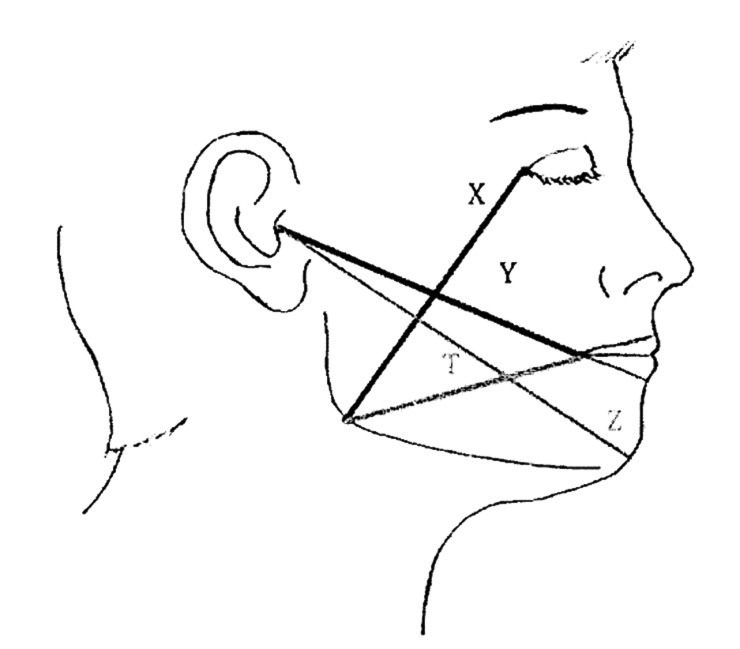
Scheme for facial swelling measurements. Line X: From the external canthus to the angle of the mandible (gonion). Line Y: From the tragus of the ear to the corner of the mouth. Line Z: From the tragus of the ear to the soft tissue pogonion. Line T: From the gonion to the corner of the mouth (52).

Variables The predictor variable was the treatment group assignment, defined as receiving photobiomodulation therapy (PBM) or no PBM following mandibular third molar surgery. All centers used the same laser device (K-Laser - multi-wavelength diode system), set with the same parameters for PBM administration. The PBM protocol was performed by the second operator on day 0, 1 and 2 using both intra-oral and extra-oral application at each session. Extra-oral setting: A specific handpiece was kept in contact with the skin on the mandibular angle (masseter insertion). The setting includes 3 phases: - Phase 1: 1.1 W peak (0.5 W at 445nm, 0.5 W at 970nm, 0.1 W at 660nm), 550 mW average power, 4 Hz, 60 seconds, 5cm2; - Phase 2: 1.1 W peak (0.5 W at 445nm, 0.5 W at 970nm, 0.1 W at 660nm), 550 mW average power, 10 Hz, 60 seconds, 5 cm2; - Phase 3: 1.1 W peak (0.5 W at 445nm, 0.5 W at 970nm, 0.1 W at 660nm), 550 mW average power, 100 Hz, 60 seconds, 5cm2. Intra-oral setting: Delivered by a specific intra-oral handpiece maintaining a distance of approximately 1 cm from the surgical suture - Phase 1: 0.4 W peak (0.1 W at 445nm, 0.2 W at 970nm, 0.1 W at 660nm), 200 mW average power, 4 Hz, 60 seconds, 2cm2 - Phase 2: 0.4 W peak (0.1 W at 445nm, 0.2 W at 970nm, 0.1 W at 660nm), 200 mW average power, 10 Hz, 60 seconds, 2cm2 - Phase 3: 0.4 W peak (0.1 W at 445nm, 0.2 W at 970nm, 0.1 W at 660nm), 200 mW average power, 100 Hz, 60 seconds, 2cm2 Outcomes Postoperative variables included pain, Health-Related Quality of Life (HR-QoL), analgesic consumption, facial edema, trismus, intraoral and extraoral hematomas, and complications such as bleeding and paresthesia. Pain intensity was assessed using a Numeric Rating Scale (NRS) on postoperative days 0, 1, 3 and 7. Health-Related Quality of Life (HRQoL) was assessed using a modified Italian translation of the questionnaire proposed by Omer Waleed Majid, translated into Italian (28). It included domains such as social isolation, work limitations, eating and diet, speech, sleep disturbances, and concerns about appearance. Each item was rated on a 4-point Likert scale (0=never to 4=very much), with domain scores ranging from 0 to 45. Patients completed the questionnaire on Day 7. A high total score indicates a high impact of the surgical intervention on the patient's quality of life. All patients were prescribed ibuprofen 600 mg sachets, and the number of sachets consumed was recorded on Day 7. Facial swelling was assessed using the linear measurement method described by Amarillas-Escobar et al. (29), based on four specific distances. Measurements were performed with a flexible measuring tape on Day 0 (preoperatively), and on postoperative Days 2 and 7, after marking five cephalometric reference points on the patient's face: The tragus, soft tissue pogonion, external canthus of the eye, gonion, and labial commissure. The four recorded linear distances were as follows: X (distance canthus-gonion); Y (trago-commissure distance); Z (trago- soft tissue pogonion distance); T (distance gonion- labial commissure) (Figure 2).

2

Trismus was assessed via maximum interincisal distance using a caliper at the same time points, preoperatively and on days 2 and 7. Presence of intraoral and extraoral hematoma was also recorded on day 2 and 7. On Day 7, secondary complications such as postoperative bleeding and paresthesia of the lower lip or chin were evaluated. Covariates The covariates considered and collected in this study include preoperative and intraoperative variables. In particular, patient age, gender, the extracted tooth, and the impaction classification according to Pell &amp;amp; Gregory and Winter. The time of the surgical procedure, measured in seconds from incision to final suture, was also documented as an objective indicator of complexity and tissue trauma. Data collection methods Data collection was performed prospectively according to a standardized protocol shared across all participating centers. Preoperative data, including demographic information (age, gender) and radiographic assessment of third molar impaction according to Pell &amp;amp; Gregory and Winter classifications, were obtained from patient medical records and pre-surgical evaluations. Intraoperative data, such as the extracted tooth number (#3.8 or #4.8) and the duration of the surgical procedure (measured in seconds from incision to final suture), were recorded in real time by the assistant. Postoperative outcomes, including pain scores, swelling measurements, trismus evaluation, and quality of life questionnaires, were collected at predefined time points by blinded evaluators to minimize bias. All data were entered into a centralized electronic database with predefined data validation rules to ensure accuracy and completeness. Data analyses Sample size was determined a priori using G*Power software (version 3.1.9.7, 2020, retrieved from https://www.psychologie.hhu.de/arbeitsgruppen/allgemeine-psychologie-undarbeitspsychologie/gpower). The calculation was based on a two-independent group comparison, assuming a significance level () of 0.05, a statistical power of 95% (1-=0.95), and a minimum effect size of Cohen's d=0.8, considered clinically meaningful. Under these parameters, the estimated number of participants required to detect a significant difference between groups was 84 (42 for each group). Although six centers were initially planned for recruitment, one withdrew before the study began, resulting in a total of five participating centers. While the initial sample size calculation indicated that 84 participants would be required to achieve 95% power (Cohen's d=0.8, =0.05), the final sample comprised 79 individuals. Under the same assumptions, this sample size (79) yields a slightly lower power, estimated at approximately 94%, which still ensures adequate sensitivity. The minimal deviation from the planned sample size does not compromise the statistical validity of the analysis. Patient were allocated using a balanced block randomization scheme stratified by study center, generated with WinPEPI software (version 11.46, retrieved from http://www.brixtonhealth.com/pepi4windows.html). Group assignments were concealed in sealed, opaque envelopes. Statistical analyses were carried out using Jamovi (version 2.6.44; The Jamovi Project, 2024, retrieved from https://www.jamovi.org/), an open-source graphical interface built on the R statistical computing environment (R Core Team, 2025, https://www.r-project.org/). Descriptive statistics were used to summarize the characteristics of the dataset. For continuous data, measures of central tendency and dispersion were calculated, including mean, median, standard deviation, minimum, and maximum; 95% confidence intervals for the means were also reported. Categorical data were summarized using absolute frequencies and relative percentages. The distribution of continuous variables was assessed using the Shapiro-Wilk test for normality, while Levene's test was applied to evaluate homogeneity of variances. Depending on the outcome of these assumptions, appropriate inferential tests were selected. Student's t-test was employed for comparisons between groups when normality and equal variance assumptions were met, whereas the non-parametric Mann-Whitney U test was used in cases where these assumptions were violated. For categorical variables, group comparisons were conducted using the chi-square test or Fisher's exact test, as appropriate. All statistical tests were two-tailed, and results were considered statistically significant at a p-value&amp;lt;0.05. Graphical displays included histograms and boxplots to examine data distributions, as well as mean plots with 95% confidence intervals to visually support group comparisons. No interim analyses were performed. The statistical analysis plan is available upon reasonable request from the corresponding author.

## Results

In the test group (TG), 12 patients were male (30.0%) and 28 were female (70.0%), with a mean age of 27.6 years (range: 20-52). In the control group (CG), 18 patients were male (48.8%) and 21 were female (51.2%), with a mean age of 26.6 years (range: 19-37). A total of 38 extracted teeth were tooth #3.8 and 41 were tooth #4.8. Each tooth was classified according to Pell and Gregory and Winter's classifications. Mean surgical time was 1831 seconds in the test group (range: 523-3960 seconds; SD: 913.5 seconds) and 1698 seconds in the control group (range: 529-4820 seconds; SD: 948.4 seconds). Both parametric (Student's t-test) and non-parametric (Mann-Whitney U) analyses showed no statistically significant difference in surgical duration between groups. Table 1 summarizes demographic and surgical characteristics of the test and control groups, including age, gender, extracted tooth, impaction classifications (Pell &amp;amp; Gregory, Winter), and surgical time.

Table 1

**Figure 3:**
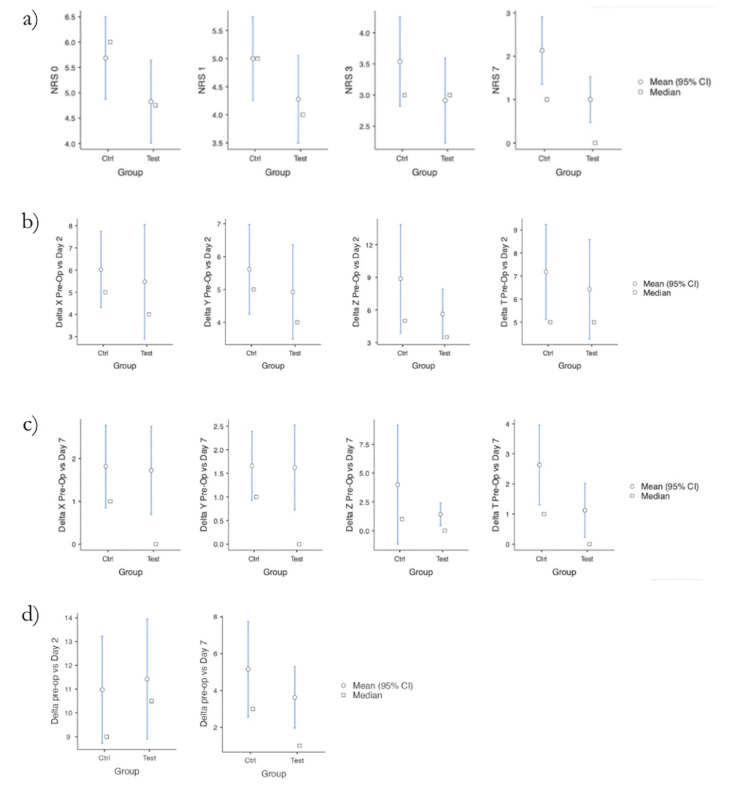
a) Mean ± 95% CI and median values of NRS scores at different time points (Days 0, 1, 3, and 7) for the Control and Test groups. b) Mean ± 95%CI and median changes in facial swelling (ΔX, ΔY, ΔZ, ΔT) from baseline to Day 2 for Control and Test groups. c) Mean ±95% CI and median changes in facial swelling (ΔX, ΔY, ΔZ, ΔT) from baseline to Day 7 for Control and Test groups. d) Mean ±95% CI and median changes in maximal interincisal distance (trismus) from baseline to Day 2 and Day 7 for Control and Test groups.

Regarding pain intensity (NRS), the test group showed lower mean scores at all time points. On day 0, the mean NRS score was 5.684 in the control group and 4.825 in the test group, with 9 and 10 patients, respectively, reporting severe to excruciating pain (NRS7). On days 1, 3, and 7 the number of patients reporting severe pain progressively decreased in both groups, remaining consistently lower in the test group. However, differences were not statistically significant on day 0, 1, or 3 (p=0.148, 0.191 and 0.218, respectively; Student's t-test). A statistically significant reduction in pain was observed on day 7 (p=0.019) in the test group (Figure 3a and Table 2).

3

Table 2

Facial swelling, evaluated along the X, Y, Z and T reference lines, was comparable between groups at both time points. On day 2, no significant differences were found (t-test; X: p=0.681, Y: p=0.496, Z: p=0.246, T: p=0.584) (Figure 3b and Table 3).

Table 3

Similarly, no significant differences were observed on day 7 (X: p=0.900, Y: p=0.920, Z: p=0.328, T: p=0.098) (Table 4 and Figure 3c).

Table 4

Trismus, assessed by interincisal distance, showed a mean reduction in mouth opening of 10.97 mm in the control group and 11.43mm in the test group on day 2. By day 7, mean reductions were 4.11mm and 3.63mm, respectively. No statistically significant differences were found at either time point (day 2: p=0.795; day 7: p=0.684), as confirmed by both Student's t-test and Mann-Whitney U test (p=0.957 and p=0.245, respectively) (Table 5 and Figure 3d).

Table 5

**Figure 4:**
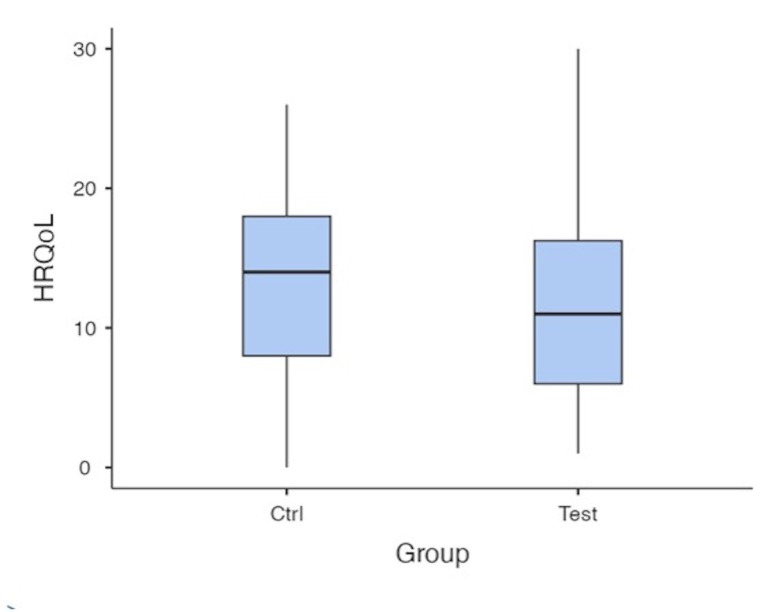
Boxplot of Health-Related Quality of Life (HRQoL) scores. Both Control and Test groups show similar distributions, with a slightly higher median in the Control group.

Mean Health-Related Quality of Life (HRQoL) scores were slightly higher in the control group (13.84±5.92) than in the test group (11.85±7.28). Nevertheless, but the difference was not statistically significant (p=0.190). The Mann-Whitney U test confirmed these findings (U=614.5, p=0.146). The effect size was small (Cohen's d=0.2993), indicating no clinically meaningful difference between the groups (Figure 4).

4

There were no significant differences in hematoma occurrence between groups. On postoperative day 2, intraoral hematomas were observed in 28.21% of patients in the control group and 27.50% in the test group, while extraoral hematoma occurred in 35.9% and 22.5% of patients, respectively. By day 7, the incidence had decreased to 17.95% (control) and 7.50% (test) for intraoral hematomas, and to 17.95% and 12.50% for extraoral hematomas. However, chi-square analysis revealed no significant association between group allocation and hematoma occurrence at either time point (intraoral: Day 2: ²=0.0049, p=0.944; day 7: ²=1.950, p=0.163; extraoral: Day 2: ²=1.717, p=0.190; day 7: ²=0.455, p=0.500). Postoperative bleeding was minimal: 73 patients reported no bleeding, and 6 experienced mild bleeding, equally distributed between groups (3 per group). Lip paresthesia occurred in three patients (1 in the control group and 2 in the test group). All cases were treated with systemic corticosteroid therapy and resolved completely within one month postoperatively.

## Discussion

Photobiomodulation (also known as Low-Level Laser Therapy), is a non-invasive technique that uses low-power light energy, typically below 500 mW, to stimulate cellular activity and promote tissue regeneration. Several studies have demonstrated that PBM protocols can reduce postoperative pain, edema, and trismus following surgical procedures, including third-molar extraction (30 , 31). Unlike surgical lasers, PBM operates at much lower energy levels and exerts anti-inflammatory, analgesic, antibacterial, and regenerative effects (1). Biologically, PBM primarily acts by stimulating mitochondria, particularly the enzyme cytochrome C oxidase (33). This enzyme, located in the mitochondrial respiratory chain, contains four active redox centers (CuA, CuB, heme a, and heme a3), making it responsive to visible and near-infrared light, especially within the 600 to 810 nm range. The absorption of light by CCO leads to increased mitochondrial activity, resulting in higher production of ATP, nitric oxide (NO), calcium ions (Ca2+), and reactive oxygen species (ROS) (34). These biochemical changes trigger cell proliferation and differentiation, as well as the activation of transcription factors involved in tissue healing and regeneration. PBM accelerates wound repair by stimulating neoangiogenesis, collagen synthesis, and cellular adhesion, while inhibiting apoptosis and promoting metabolic activity through enhanced ATP production (35). It also activates mononuclear cells via leukotriene B4 and increases interleukin-8 levels, facilitating fibroblast proliferation and supporting soft tissue repair (36). In the context of hard tissue healing, PBMT has been shown to enhance chondroblast proliferation, osteogenesis, and bone differentiation, with increased expression of osteocalcin (37). The analgesic effect of PBM is attributed to both direct and indirect mechanisms. On one hand, it inhibits pain transmission by hyperpolarizing axonal membranes and blocking signal propagation along A and C nerve fibers. On the other, it modulates inflammatory pathways and promotes the release of endogenous endorphins, thereby elevating the pain threshold similar to opioid mechanisms (38). Furthermore, PBM exhibits antibacterial properties that vary depending on the laser's wavelength, power output, pulse duration, and emission mode, as well as on bacterial characteristics such as pigmentation and cell wall structure (39). The most frequently proposed mechanism is photothermal, with bacterial inactivation resulting from heat generation rather than a specific photochemical reaction. Therapeutic outcome of PBM depend strongly on laser parameters including wavelength, power output, energy density (fluence), frequency, and exposure time. In our study, the protocol involved the use of a multiwavelength diode laser system, combining visible wavelengths (445nm and 660nm) with near-infrared light (970nm), with the aim of simultaneously targeting multiple biological chromophores and mechanisms. This multi-spectral approach was designed to exploit the distinct tissue penetration depths and photobiological effects associated with each wavelength, thereby maximizing the potential therapeutic benefits. The visible wavelengths are primarily absorbed by superficial tissues and specific cellular components, promoting processes such as cellular proliferation and collagen synthesis, while the near-infrared wavelength penetrates deeper, stimulating mitochondrial activity and enhancing tissue regeneration at greater depths (40). The fluence (energy density, expressed in J/cm²) plays a critical role in therapeutic outcome. PBM follows a i biphasic dose-response curve (Arndt-Schulz law (41)), in which low fluence fails to elicit a response while excessive fluence inhibits it (42). For superficial pathologies, energy densities in the range of 1-10 J/cm² are commonly used, while higher doses (10-50 J/cm²) are applied for deeper conditions treated with NIR wavelengths. Notably, the same fluence applied over different surface areas can result in vastly different total energy delivery, potentially affecting the outcome. Despite its theoretical advantages and safety profile, consistently described as well-tolerated and free from adverse effects, clinical studies investigating PBM in third molar surgery report controversial outcomes. Campos et al. performed a systematic review and meta-analysis to evaluate the effectiveness of PBM for pain control following third molar extraction (31). Their analysis, which included eight randomized controlled trials with a total of 498 patients, showed that PBMT significantly reduced postoperative pain on day two, although this effect was no longer significant by day seven. The authors also highlighted a high degree of heterogeneity among the included studies, particularly in terms of wavelengths (most commonly 810 nm), application techniques, session frequency, and dosimetry. Similarly, Domah et al. (43) analyzed seventeen RCTs and found a significant reduction in postoperative swelling on days 2 and 7 (SMD= -0.611 and -0.532, respectively) in patients treated with LLLT compared to placebo. However, no statistically significant effects were observed regarding pain or trismus. Oliveira et al. also reported favorable results with infrared wavelengths (780-904nm), attributing the effectiveness to superior tissue penetration (44). Aras et al. demonstrated that intraoral PBMT was effective in reducing trismus by day 7, while extraoral PBMT reduced swelling as early as day 2 (8). Further supporting these observations, Eshghpour et al. (45) performed a randomized, double-blind, split-mouth trial on 40 patients with symmetrically impacted mandibular third molars. The study employed a combined protocol involving intraoral irradiation with a 660nm laser and extraoral application with an 810nm laser, repeated on postoperative days 2 and 4. Their results revealed statistically significant reductions in both pain and swelling at all assessed time points (p&amp;lt;.05), reinforcing the potential utility of LLLT in managing postoperative discomfort. Additional evidence comes from Camolesi et al. (46), who conducted a double center randomized clinical trial to evaluate the efficacy of photobiomodulation therapy (PBMT) on postoperative pain, facial swelling, and trismus following bilateral mandibular third molar extraction. Eighty-three patients underwent extractions, with one side receiving active PBMT and the other a placebo simulation. The intervention employed an 808nm, 100mW Ga-Al-As diode laser, delivering 3J per point in a single postoperative session applied intraorally and extraorally. Pain, edema, and trismus were recorded at multiple time points, and analgesic consumption was monitored over seven days. The PBMT group showed significantly reduced pain, edema, and trismus on postoperative days 2 and 7 (p&amp;lt;.01), along with decreased analgesic use on all days except day 7. These results support the clinical effectiveness of a single-session PBMT protocol in reducing postoperative morbidity associated with third molar surgery. Similarly, González et al. (47) conducted a systematic review and meta-analysis of 18 split-mouth randomized clinical trials evaluating the effects of Low-Level Laser Therapy (LLLT) on postoperative outcomes following third molar extraction. The results indicated a potential benefit of LLLT in reducing postoperative pain at 48 hours (SMD, -1.11; 95% CI, -1.63 to -0.58; p&amp;lt;.001), swelling at 48 hours (SMD, -0.50; 95% CI, -0.88 to -0.11; p=.01), and trismus at 7 days (SMD, 0.35; 95% CI, 0.16 to 0.53; p=.002). Despite these findings, the authors emphasized that the certainty of the evidence remains low, highlighting the need for further high-quality randomized clinical trials. Conversely, other studies have less robust results. Eroglu and Tunc (48) investigated the effects of a single extraoral application of LLLT using a 940nm diode laser at 4J/cm² in 35 patients undergoing bilateral extraction of similarly impacted third molars. Although no statistically significant differences in pain, swelling, or trismus were detected between the laser-treated and placebo sides (Mann-Whitney U test, p&amp;gt;0.05), the laser group demonstrated a tendency toward reduced swelling and trismus. Similarly, Uzeda et al. (49) conducted a randomized controlled trial evaluating the effects of Low-Level Laser Therapy (LLLT) at 660nm and 808nm wavelengths on postoperative outcomes following mandibular third molar extraction. Thirty patients were randomly assigned to three groups-control, 660nm, and 808nm and received laser irradiation both before and immediately after surgery. Pain, swelling, and trismus were measured preoperatively, immediately postoperatively, and on day 7. Although no statistically significant differences were found among groups (p&amp;gt;0.05), both laser-treated groups exhibited lower subjective pain perception compared to the control, suggesting a possible benefit of LLLT in reducing postoperative discomfort. Although most research has concentrated on postoperative LLLT application, some authors have proposed preoperative use to proactively modulate the inflammatory response. For example, Karc and Balaban (50) assessed the effects of a single LLLT session administered 10 minutes prior to surgery. While no significant differences were found between the laser and control groups in terms of pain, swelling, or trismus, the clinical results were deemed acceptable, suggesting potential benefit of preoperative application. In the present randomized controlled trial, we evaluated the clinical effectiveness of PBM in reducing postoperative complications following mandibular third-molar surgery. No statistically significant differences between the PBM and control groups for any outcome measured. Both parametric and non-parametric analyses confirmed that pain intensity, facial swelling, trismus, HRQoL, hematoma occurrence, surgical time, and postoperative bleeding were comparable at all time points. Although mean pain scores in the PBM group were consistently lower, these differences did not reach statistical significance (e.g., day 0: p=0.148; day 3: p=0.218). Similarly, measurements of facial swelling at days 2 and 7 (across X, Y, Z, and T lines) yielded p-values above 0.05. Nonetheless, some variables demonstrated favorable trends in the PBM group, especially regarding pain perception, swelling, trismus, and quality of life. These trends suggest a potential therapeutic benefit that may have been underestimated due to limited statistical power. Small to moderate effect sizes were observed; however, our sample size was likely insufficient to detect these differences. Power analysis confirmed this limitation. For example, detecting an effect size for pain reduction on day 0 (Cohen's d 0.33) would have required at least 143 participants for 80% power at =0.05. Similarly, identifying differences in HRQoL would have necessitated approximately 176 subjects. The effect sizes for facial swelling were especially small (Cohen's d&amp;lt;0.1), indicating that detecting such subtle changes would require very large sample sizes, potentially without clinical relevance. To ensure consistency in surgical execution, all procedures were performed by the same experienced surgeon. Outcome evaluation was conducted by a blinded examiner to minimize bias, particularly in facial measurements. Although a split-mouth model could have minimized inter-individual variability by allowing each patient to serve as their own control, this approach was not adopted in our study. The decision was based on the fact that the left and right mandibular third molars often present substantial anatomical and surgical differences-such as variations in impaction depth, angulation, root morphology, and proximity to neurovascular structures as well as preference of the operator, according to his/her preferred side of work. These discrepancies could lead to inherently different surgical complexities and postoperative outcomes, thereby introducing bias and reducing the internal validity of the study.

## Conclusions

In conclusion, while our study did not demonstrate statistically significant differences between PBMT and control groups (except for pain on day 7 with benefit of test group versus control group), the observed trends confirm potential clinical benefits in all the analyzed variables. These preliminary findings warrant further investigation through larger, adequately powered trials with standardized protocols to definitively establish the role of PBMT in managing postoperative outcomes after third molar surgery.

## Figures and Tables

**Table 1:** Table Comparative analysis between test and control group for age, gender, extracted tooth, Pell &amp; Gregory and Winter's classifications, and surgical time.

Variable	Test Group (TG, n=40)	Control Group (CG, n=39)	p-value
Age (years), mean±SD	27.6±6.63 (20-52)	26.6±4.4 (19â37)	0.429
Gender (M/F)	12 (30%)/28 (70%)	18 (48.8%)/21 (51.2%)	0.1681
Extracted tooth (#3.8/#4.8)	18 (45%)/22 (55%)	20 (51.3%)/19 (48.7%)	0.6551
Pell &amp; Gregory Class A/B/C	20/15/5	18/16/5	*
Winter classification (Mesio/Vertical/Horizontal)	22/10/08	21/11/07	*
Surgical time (sec), mean±SD	1831±913.5	1698±948.4	0.528

*Not applicable condition; 1p-values calculated by Fisher's exact test.

**Table 2:** Table Comparison between groups using Independent Samples T-Test, Bayes Factor and Mann-Whitney U across different variables (NRS 0,1,3,7).

Covariates		Statistic	±%	df	p	Mean difference	SE difference	Effect Size
NRS 0	Studentâs t	1.46		76.0	0.148	0.859	0.588	Cohenâs d 0.331
	Bayes factor10	0.589	1.33eâ4					Rank biserial â0.215
	MannâWhitney U	597			0.101	1.000		
NRS 1	Studentâs t	1.32		76.0	0.191	0.725	0.550	Cohenâs d 0.299
	Bayes factor10	0.497	1.39eâ4					Rank biserial â0.163
	MannâWhitney U	637			0.215	1.000		
NRS 3	Studentâs t	1.24		77.0	0.218	0.626	0.504	Cohenâs d 0.279
	Bayes factor10	0.455	1.44eâ4					Rank biserial â0.152
	MannâWhitney U	662			0.242	1.000		
NRS 7	Studentâs t	2.39áµ		76.0	0.019	1.132	0.474	Cohenâs d 0.541
	Bayes factor10	2.657	8.57eâ5					Rank biserial â0.320
	MannâWhitney U	517			0.010	1.000		

H μctrl ≠ μtest. Levene's test is significant (p&lt;.05).

**Table 3:** Table Results of Independent Samples t-tests, Mann-Whitney U tests, and Bayes factor analyses comparing changes in facial swelling (ΔX, ΔY, ΔZ, ΔT) from pre-operative baseline to Day 2 between Control and Test groups. No statistically significant differences were observed between groups for any swelling dimension (all p&gt;0.05).

Covariates		Statistic	±%	df	p	Mean difference	SE difference	Effect Size
ÎX (pre-op vs Day 2)	Studentâs t	0.412		77.0	0.681	0.653	1.58	Cohenâs d 0.093
	Bayes factor10	0.252	1.68eâ4					
	MannâWhitney U	654			0.214	1.000		Rank biserial â0.162
ÎY (pre-op vs Day 2)	Studentâs t	0.685		77.0	0.496	0.690	1.01	Cohenâs d 0.154
	Bayes factor10	0.286	1.68eâ4					
	MannâWhitney U	671			0.284	1.000		Rank biserial â0.140
ÎZ (pre-op vs Day 2)	Studentâs t	1.169		77.0	0.246	3.247	2.78	Cohenâs d 0.263
	Bayes factor10	0.422	1.47eâ4					
	MannâWhitney U	607			0.088	2.000		Rank biserial â0.222
ÎT (pre-op vs Day 2)	Studentâs t	0.550		77.0	0.584	0.831	1.51	Cohenâs d 0.124
	Bayes factor10	0.266	1.65eâ4					
	MannâWhitney U	682			0.336	1.000		Rank biserial â0.126

H μctrl ≠ μtest.

**Table 4:** Table Results of Independent Samples t-tests, Mann-Whitney U tests, and Bayes factor analyses comparing changes in facial swelling (ΔX, ΔY, ΔZ, ΔT) from pre-operative baseline to Day 7 between Control and Test groups. No statistically significant differences were found between groups (all p&gt;0.05).

Covariates		Statistic	±%	df	p	Mean difference	SE difference	Effect Size
ÎX (pre-op vs Day 7)	Studentâs t	0.126		76.0	0.900	0.091	0.722	Cohenâs d 0.029
	Bayes factor10	0.237	1.67eâ4					
	MannâWhitney U	644			0.210	5.44e-5		Rank biserial â0.153
ÎY (pre-op vs Day 7)	Studentâs t	0.100		76.0	0.920	0.059	0.591	Cohenâs d 0.023
	Bayes factor10	0.236	1.67eâ4					
	MannâWhitney U	661			0.296	4.53e-5		Rank biserial â0.131
ÎZ (pre-op vs Day 7)	Studentâs t	0.985		76.0	0.328	2.578	2.617	Cohenâs d 0.223
	Bayes factor10	0.358	1.51eâ4					
	MannâWhitney U	656			0.255	5.35e-5		Rank biserial â0.137
ÎT (pre-op vs Day 7)	Studentâs t	1.673		76.0	0.098	1.329	0.794	Cohenâs d 0.379
	Bayes factor10	0.782	1.24eâ4					
	MannâWhitney U	519			0.009	1.000		Rank biserial â0.317

H μctrl ≠ μtest.

**Table 5:** Table Results of Independent Samples t-tests, Mann-Whitney U tests, and Bayes factor analyses comparing changes in maximal interincisal distance (trismus) between Control and Test groups from pre-operative baseline to Day 2 and Day 7. No statistically significant differences were found between groups at either time point (p=0.795 for Day 2; p=0.684 for Day 7).

Covariates		Statistic	±%	df	p	Mean difference	SE difference
Î pre-op vs Day 2	Studentâs t	â0.261		77.0	0.795	â0.451	1.73
	Bayes factor10	0.241	1.70eâ4				
	MannâWhitney U	774			0.957	-2.73e-5	
Î pre-op vs Day 7	Studentâs t	0.408		76.0	0.684	0.480	1.18
	Bayes factor10	0.253	1.64eâ4				
	MannâWhitney U	647			0.245	3.15e-5	

H μctrl ≠ μtest.

## Data Availability

Declared none.
